# Risk Factors of Inadequate Bowel Preparation for Screening Colonoscopy

**DOI:** 10.3390/jcm10122740

**Published:** 2021-06-21

**Authors:** Efrat L. Amitay, Tobias Niedermaier, Anton Gies, Michael Hoffmeister, Hermann Brenner

**Affiliations:** 1 Division of Clinical Epidemiology and Aging Research, German Cancer Research Center (DKFZ), 69120 Heidelberg, Germany; t.niedermaier@dkfz-heidelberg.de (T.N.); m.hoffmeister@dkfz-heidelberg.de (M.H.); h.brenner@dkfz.de (H.B.); 2 Division of Preventive Oncology, German Cancer Research Center (DKFZ) and National Center for Tumor Diseases (NCT), 69120 Heidelberg, Germany; anton.gies@nct-heidelberg.de; 3 German Cancer Consortium (DKTK), German Cancer Research Center (DKFZ), 69120 Heidelberg, Germany

**Keywords:** colorectal neoplasms, colonoscopy, cancer screening, bowel preparation

## Abstract

The success of a colonoscopy in detecting and removing pre-cancerous and cancerous lesions depends heavily on the quality of bowel preparation. Despite efforts, 20–44% of colonoscopy participants have an inadequate bowel preparation. We aimed to assess and compare risk factors for inadequate bowel preparation and for the presence of advanced colorectal neoplasms in routine screening practice. In this cross-sectional study, among 8125 participants of screening colonoscopy in Germany with a comprehensive assessment of sociodemographic factors, lifestyle and medical history, we examined factors associated with inadequate bowel preparation and with findings of advanced neoplasms using adjusted log-binomial regression models. Among the identified risk factors assessed, three factors were identified that were significantly associated with inadequate bowel preparation: age ≥ 70 years (adjusted prevalence ratios, aPR, 1.50 95%CI 1.31–1.71), smoking (aPR 1.29 95%CI 1.11–1.50) and abdominal symptoms (aPR 1.14 95%CI 1.02–1.27). The same risk factors were also associated with the prevalence of advanced neoplasms in our study (aPR 1.72, 1.62 and 1.44, respectively). The risk factors associated with inadequate bowel preparation in this study were also associated with a higher risk for advanced neoplasms. Inadequate bowel preparation for colonoscopy might lead to missed colorectal cancer (CRC) precursors and the late diagnosis of CRC. People at high risk of advanced neoplasms are in particular need of enhanced bowel preparation.

## 1. Introduction

Colorectal cancer (CRC) is the third most commonly diagnosed malignancy and the second most common cause of cancer-related death worldwide. In 2018, CRC accounted for over 1.8 million incident cases and almost 900,000 deaths [1].

Colonoscopy, conducted either for primary screening or as a follow-up for fecal occult blood tests, is the gold standard in early detection of CRC. Screening colonoscopy has the potential to prevent a very large share of CRC incidence and mortality by detecting and removing precursors of the disease [2,3,4]. The ability to detect precursors strongly depends on the quality of bowel preparation, which facilitates the clear visualization of the mucosal surface [5]. Despite efforts to facilitate the process, 20–44% of colonoscopy participants have been reported in previous studies to have inadequate bowel preparation [6,7,8].

Inadequate bowel preparation may lead to incomplete colonoscopies [9] and precancerous lesions being missed and may necessitate repeat colonoscopy exams, a burden both to patients and the health system. The ability to identify factors associated with inadequate bowel preparation may help in targeting efforts to increase adequate preparation towards populations at higher risk. Therefore, we aimed to assess factors associated with the quality of bowel preparation for colonoscopy in a large routine screening colonoscopy cohort in Germany.

## 2. Materials and Methods

Our analysis is based on participants from KolosSal, a large German CRC screening cohort which has been described in detail elsewhere [10]. Briefly, the primary aim of KolosSal was monitoring the long-term reduction in CRC incidence and mortality among participants of screening colonoscopy, which has been offered in Germany since 2002. Overall, 19,177 participants of screening colonoscopy were recruited in gastroenterological practices in the German state of Saarland during 2005–2013 and completed a short questionnaire. Information on the quality of bowel preparation and findings at colonoscopy were extracted from colonoscopy records. The study was approved by the ethics committees of the medical faculty of the Heidelberg University and the Saarland Medical Association, and written informed consent was obtained from each participant.

The current analysis was restricted to 8125 participants with no colonoscopy in the previous year for whom the quality of bowel preparation was explicitly reported in the colonoscopy record (*n* = 279 participants with a previous colonoscopy in the past year were excluded). Information on bowel preparation and findings at colonoscopy were reported by clinicians as free text and extracted in a standardized manner. Whenever the report included remarks on impaired cleanliness of the bowel, i.e., impaired visibility or obstruction due to stool, or an incomplete colonoscopy due to insufficient bowel cleanliness, bowel preparation was classified as inadequate.

Based on such information, the quality of bowel preparation was classified as adequate or inadequate. Participants with adequate and inadequate bowel preparation were compared with respect to factors known or suspected to be associated with CRC risk: sex, age at colonoscopy, body mass index (BMI), education, smoking, alcohol consumption, red and processed meat consumption, aspirin use, past large bowel endoscopy, hypertension, diabetes and abdominal symptoms prior to colonoscopy. Log-binomial regression was used to quantify the independent association of those factors with (i) the quality of bowel preparation and (ii) the detection of at least one advanced neoplasm (CRC or advanced adenomas; thus, defined if they matched any of the following criteria: size ≥ 1 cm, villous or tubulo-villous architecture or high-grade dysplasia). The analysis of associations with findings of advanced neoplasms was restricted to those with reported adequate bowel preparation to minimize potential bias by missed neoplasms.

Analyses were conducted in R 3.6.1 [11] and ten multiple imputations were performed using the “mice” package to deal with missing values in exposure variables (0–16% missing). Statistical significance was defined by a *p*-value of *p* < 0.05 in 2-sided testing.

## 3. Results

Table 1 shows main characteristics of the study population and their associations with the quality of bowel preparation. Overall, 8125 study participants were eligible for analysis. Women accounted for 49.5% of participants and the mean age was 63.3 ± 6.9 years. Slightly more than half of the participants (52.1%) smoked in the past or were smoking at the time of joining the study and for 70.4% it was their first colonoscopy.

As seen in Table 1, inadequate bowel preparation was significantly more often reported for participants ≥ 70 years of age (22.5%), current smokers (20.4%), regular users of aspirin (19.5%), participants with diabetes (20.5%) and participants who reported abdominal symptoms before colonoscopy (19.9%). In multivariate adjusted analyses, statistically significant prevalence ratios (aPRs, 95% CI) were found for participants ≥ 70 years of age (aPR = 1.50, 95%CI 1.31–1.71), current smokers (aPR = 1.29, 95%CI 1.11–1.50) and those reporting abdominal symptoms before colonoscopy (aPR = 1.14, 95%CI 1.02–1.27).

Table 2 presents the risk factors for advanced neoplasms among study participants with adequate bowel preparation. These same factors, which were found to be associated with inadequate bowel preparation, were also found to be strongly associated with findings of advanced neoplasms among those with adequate bowel preparation, with aPR = 1.72 (95%CI 1.42–2.08), aPR = 1.62 (95%CI 1.31–2.00) and aPR = 1.44 (95%CI 1.24–1.68), for age ≥ 70 years, current smoking and abdominal symptoms, respectively.

## 4. Discussion

In this study, among more than 8000 participants of screening colonoscopy, some of the strongest risk factors for the presence of CRC, i.e., old age, smoking and abdominal symptoms, were suggested to also be risk factors for inadequate bowel preparation. Since poor bowel preparation goes along with an increased risk of missed relevant colorectal neoplasms [9] these results underline the importance of efforts to ensure the best possible bowel preparation in routine screening practice.

A number of previous, mostly smaller, studies have assessed the prevalence and risk factors of poor bowel preparation in screening and clinical settings as shown in Supplementary Table S1 [8,12,13,14,15]. The association of inadequate bowel preparation with older age may be explained by a worse mental and functional state. In one study, failed colonoscopy among nonagenarians was due to a bad preparation which was inversely associated with the mental and functional state [16]. Abdominal symptoms may be due to anatomical reason or medical disorders that lead to constipation or obstructions in the bowel which were found to be associated with inadequate bowel preparation due to infrequent bowel movements [17].

To our knowledge, ours is the largest study that investigated the role of risk factors for poor bowel preparation exclusively in an outpatient routine screening setting, and the first one to asses several risk factors of inadequate bowel preparation and of advanced colorectal neoplasms side by side in the same study population. In order to minimize bias from missed advanced neoplasms, the latter association was assessed among participants for whom adequate bowel preparation was explicitly documented.

Our results are based on a questionnaire and colonoscopy data collected from participants of a German population-based CRC screening colonoscopy program. With 8125 participants, it is the largest study to date to investigate factors associated with inadequate bowel preparation for colonoscopy conducted solely in a population screening setting. Since adequate bowel cleaning and completed colonoscopy rates differ between inpatient and screening setting [18], our study offers a more accurate view on groups that may need more guidance in preparing for colonoscopies in a population screening setting.

Our study also has limitations. The study included participants who not only chose to attend screening colonoscopy, but also to participate in the study. Most likely populations that tend to have lower participation rates in screening programs such as those with language barriers or from lower socio-economic backgrounds will be underrepresented [19]. Possibly, these groups might also have greater difficulties in performing bowel cleaning properly according to instructions; thus, possibly leading to an even higher rate of inadequate bowel preparation. The information is based on self-administered questionnaires.

Another limitation of our study is that the quality of bowel preparation was reported in approximately half of the colonoscopy reports only and was not rated by an established score. The latter may have led to an imperfect classification of the quality of bowel preparation and, as a result, to some underestimation of associations. On the other hand, our results may well reflect routine screening practice outside academic centers where most screening colonoscopies are conducted and standardized documentation of the quality of bowel preparation is not established.

In conclusion, our study suggests that risk factors for advanced neoplasms such as older age, smoking and having ongoing abdominal symptoms are also risk factors for inadequate bowel preparation for colonoscopy, possibly leading to missed CRC precursors during screening colonoscopy. More efforts should be made to better identify and prepare these higher risk populations adequately for screening colonoscopies.

## Figures and Tables

**Table 1:** Characteristics of the study population, association with inadequate bowel preparation and risk of advanced neoplasms.

Characteristics		Prevalence Ratio (95% CI)					
	Total	Inadequate Bowel Preparation	Risk Factors for Inadequate Bowel Preparation		Advanced Neoplasms	Risk Factors for Advanced Neoplasms	
	*n*	*n* (Row %)	Crude	Adjusted *	*n* (Row %)	Crude	Adjusted *
Sex							
Women	4023	687 (17.1)	Reference	Reference	317 (7.9)	Reference	Reference
Men	4102	726 (17.7)	1.04 (0.94–1.14)	1.03 (0.93–1.15)	595 (14.5)	1.84 (1.62–2.09)	1.66 (1.45–1.91)
Age at colonoscopy (years)							
<60	3128	477 (15.2)	Reference	Reference	288 (9.2)	Reference	Reference
60–64	1706	296 (17.4)	1.14 (1.00–1.30)	1.14 (1.00–1.30)	177 (10.4)	1.13 (0.94–1.35)	1.14 (0.95–1.36)
65–69	1639	268 (16.4)	1.07 (0.93–1.23)	1.08 (0.94–1.24)	197 (12.0)	1.31 (1.10–1.55)	1.35 (1.14–1.61)
≥70	1652	372 (22.5)	1.48 (1.31–1.67)	1.50 (1.31–1.71)	250 (15.1)	1.64 (1.40–1.93)	1.77 (1.50–2.09)
Body Mass Index (BMI) (kg/m^2^)							
<25	2427	420 (17.3)	Reference	Reference	229 (9.4)	Reference	Reference
25–29.9	3567	585 (16.4)	0.94 (0.84–1.06)	0.94 (0.84–1.06)	402 (11.3)	1.19 (1.02–1.38)	1.05 (0.90–1.23)
30+	1832	353 (19.3)	1.11 (0.98–1.26)	1.12 (0.97–1.28)	237 (12.9)	1.37 (1.15–1.62)	1.23 (1.03–1.47)
Education (years)							
≤9	5085	929 (18.3)	Reference	Reference	929 (18.3)	Reference	Reference
10–11	1399	218 (15.6)	0.86 (0.75–0.98)	0.91 (0.79–1.04)	218 (15.6)	0.78 (0.65–0.94)	0.89 (0.74–1.07)
12+	1371	220 (16)	0.87 (0.76–1.00)	0.93 (0.81–1.07)	220 (16.0)	0.94 (0.79–1.11)	0.94 (0.79–1.12)
Smoking							
Never	3792	623 (16.4)	Reference	Reference	344 (9.1)	Reference	Reference
Past	3218	567 (17.6)	1.07 (0.96–1.18)	1.07 (0.96–1.19)	405 (12.6)	1.39 (1.22–1.59)	1.21 (1.05–1.39)
Current	912	186 (20.4)	1.24 (1.07–1.43)	1.29 (1.11–1.50)	134 (14.7)	1.63 (1.35–1.96)	1.58 (1.30–1.91)
High alcohol consumption ^+^							
No	5562	961 (17.3)	Reference	Reference	586 (10.5)	Reference	Reference
Yes	1249	212 (17)	0.98 (0.85–1.13)	0.97 (0.84–1.12)	182 (14.6)	1.38 (1.17–1.63)	1.21 (1.02–1.43)
High red and processed meat consumption ^++^							
No	7101	1218 (17.2)	Reference	Reference	767 (10.8)	Reference	Reference
Yes	760	136 (17.9)	1.04 (0.88–1.22)	1.02 (0.86–1.20)	112 (14.7)	1.36 (1.13–1.63)	1.17 (0.97–1.41)
Current regular aspirin use							
No	6499	1098 (16.9)	Reference	Reference	728 (11.2)	Reference	Reference
Yes	1383	269 (19.5)	1.15 (1.02–1.30)	1.05 (0.93–1.19)	156 (11.3)	1.00 (0.85–1.18)	0.78 (0.66–0.92)
Past endoscopy							
No	5591	956 (17.1)	Reference	Reference	710 (12.7)	Reference	Reference
Yes	2353	428 (18.2)	1.06 (0.96–1.18)	1.01 (0.91–1.12)	177 (7.5)	0.59 (0.51–0.70)	0.56 (0.47–0.65)
Hypertension							
No	4125	727 (17.6)	Reference	Reference	427 (10.4)	Reference	Reference
Yes	3741	639 (17.1)	0.97 (0.88–1.07)	0.88 (0.80–0.98)	451 (12.1)	1.16 (1.03–1.31)	1.04 (0.91–1.18)
Diabetes							
No	6820	1151 (16.9)	Reference	Reference	725 (10.6)	Reference	Reference
Yes	1058	217 (20.5)	1.21 (1.06–1.38)	1.13 (0.98–1.29)	154 (14.6)	1.37 (1.16–1.61)	1.14 (0.96–1.34)
Abdominal symptoms before colonoscopy							
No	5922	979 (16.5)	Reference	Reference	606 (10.2)	Reference	Reference
Yes	1954	388 (19.9)	1.20 (1.07–1.33)	1.14 (1.02–1.27)	268 (13.7)	1.34 (1.18–1.54)	1.40 (1.23–1.61)

* Adjusted for all other covariates listed in the column; ^+^ High alcohol consumption: regular intake of alcohol on ≥5 days a week with one standard alcoholic beverage equaling 0.33 L beer, 0.25 L wine or 2 cL hard liquor. ^++^ Defined as high if the participant consumed red and processed meat at least once per day. Abbreviations: CI—confidence interval; col%—column percent, e.g., 4023/(4023 + 4102) = 49.5% for women in variable “sex”; Row%—row percent, e.g., 687/4023 = 17.1% for inadequate bowel preparation among women.

**Table 2:** Characteristics of the study population and association with prevalence of advanced neoplasms among participants with adequate bowel preparation.

Characteristics	Participants				Prevalence Ratio (95% CI)	
	Adequate Bowel Preparation		Advanced Neoplasms		Crude	Adjusted *
	*n*	Col %	*n*	Row %		
Sex						
Women	3336	49.7	260	7.8	Reference	Reference
Men	3376	50.3	481	14.2	1.83 (1.58–2.11)	1.65 (1.41–1.92)
Age at colonoscopy (years)						
<60	2651	39.5	244	9.2	Reference	Reference
60–64	1410	21.0	152	10.8	1.17 (0.97–1.42)	1.20 (0.99–1.45)
65–69	1371	20.4	162	11.8	1.28 (1.06–1.55)	1.34 (1.11–1.62)
≥70	1280	19.1	183	14.3	1.55 (1.30–1.86)	1.72 (1.42–2.08)
Body Mass Index (BMI) (kg/m^2^)						
<25	2007	31.0	187	9.3	Reference	Reference
25–29.9	2982	46.1	330	11.1	1.19 (1.00–1.41)	1.06 (0.89–1.26)
30+	1479	22.9	186	12.6	1.36 (1.13–1.65)	1.26 (1.03–1.54)
Education (years)						
≤9	4156	64.1	475	11.4	Reference	Reference
10–11	1181	18.2	104	8.8	0.77 (0.63–0.94)	0.86 (0.71–1.05)
12+	1151	17.7	132	11.5	0.99 (0.83–1.19)	1.00 (0.83–1.20)
Smoking						
Never	3169	48.4	280	8.8	Reference	Reference
Past	2651	40.5	326	12.3	1.39 (1.19–1.61)	1.20 (1.03–1.41)
Current	726	11.1	109	15.0	1.70 (1.38–2.09)	1.62 (1.31–2.00)
High alcohol consumption ^+^						
No	4601	81.6	473	10.3	Reference	Reference
Yes	1037	18.4	151	14.6	1.42 (1.19–1.69)	1.24 (1.04–1.48)
Red and processed meat consumption						
<1 per day	5883	90.4	626	10.6	Reference	Reference
≥1 per day	624	9.6	89	14.3	1.34 (1.09–1.64)	1.16 (0.95–1.42)
Current regular aspirin use						
No	5401	82.9	598	11.1	Reference	Reference
Yes	1114	17.1	119	10.7	0.96 (0.80–1.16)	0.79 (0.65–0.95)
Past endoscopy						
No	4635	70.7	579	12.5	Reference	Reference
Yes	1925	29.3	140	7.3	0.58 (0.49–0.70)	0.55 (0.46–0.65)
Hypertension						
No	3398	52.3	352	10.4	Reference	Reference
Yes	3102	47.7	362	11.7	1.12 (0.97–1.29)	1.01 (0.87–1.17)
Diabetes						
No	5669	87.1	598	10.5	Reference	Reference
Yes	841	12.9	117	13.9	1.32 (1.10–1.59)	1.10 (0.91–1.33)
Abdominal symptoms before colonoscopy						
No	4943	75.9	496	10.0	Reference	Reference
Yes	1566	24.1	212	13.5	1.36 (1.17–1.58)	1.44 (1.24–1.68)

* Adjusted for all other covariates listed in the column; ^+^ High alcohol consumption: regular intake of alcohol on ≥5 days a week with one standard alcoholic beverage equaling 0.33 L beer, 0.25 L wine or 2 cL hard liquor. Abbreviations: CI—confidence interval; col%—column percent, e.g., 4023/(4023 + 4102) = 49.5% for women in variable “sex”; Row%—row percent, e.g., 687/4023 = 17.1% for inadequate bowel preparation among women.

## Data Availability

The data presented in this study are available on request from the corresponding author. The data are not publicly available due to privacy restrictions.

## Supplementary Materials

The following are available online at https://www.mdpi.com/article/10.3390/jcm10122740/s1, Table S1: Previous studies assessing risk factors for inadequate bowel preparation.

## References

[1] Bray F., Ferlay J., Soerjomataram I., Siegel R.L., Torre L.A., Jemal A. (2018). Global cancer statistics 2018: GLOBOCAN estimates of incidence and mortality worldwide for 36 cancers in 185 countries. CA Cancer J. Clin..

[2] Brenner H., Stock C., Hoffmeister M. (2014). Effect of screening sigmoidoscopy and screening colonoscopy on colorectal cancer incidence and mortality: Systematic review and meta-analysis of randomised controlled trials and observational studies. BMJ.

[3] Chen C., Stock C., Hoffmeister M., Brenner H. (2018). Public health impact of colonoscopy use on colorectal cancer mortality in Germany and the United States. Gastrointest. Endosc..

[4] Brenner H., Chen C. (2018). The colorectal cancer epidemic: Challenges and opportunities for primary, secondary and tertiary prevention. Br. J. Cancer.

[5] Sulz M.C., Kröger A., Prakash M., Manser C.N., Heinrich H., Misselwitz B. (2016). Meta-Analysis of the Effect of Bowel Preparation on Adenoma Detection: Early Adenomas Affected Stronger than Advanced Adenomas. PLoS ONE.

[6] Adler A., Wegscheider K., Lieberman D., Aminalai A., Aschenbeck J., Drossel R., Mayr M., Mroß M., Scheel M., Schröder A. (2013). Factors determining the quality of screening colonoscopy: A prospective study on adenoma detection rates, from 12 134 examinations (Berlin colonoscopy project 3, BECOP-3). Gut.

[7] Froehlich F., Wietlisbach V., Gonvers J.-J., Burnand B., Vader J.-P. (2005). Impact of colonic cleansing on quality and diagnostic yield of colonoscopy: The European Panel of Appropriateness of Gastrointestinal Endoscopy European multicenter study. Gastrointest. Endosc..

[8] Fayad N.F., Kahi C.J., El–Jawad K.H.A., Shin A.S., Shah S., Lane K.A., Imperiale T. (2013). Association Between Body Mass Index and Quality of Split Bowel Preparation. Clin. Gastroenterol. Hepatol..

[9] Chokshi R.V., Hovis C.E., Hollander T., Early D.S., Wang J.S. (2012). Prevalence of missed adenomas in patients with inadequate bowel preparation on screening colonoscopy. Gastrointest. Endosc..

[10] Brenner H., Haug U., Arndt V., Stegmaier C., Altenhofen L., Hoffmeister M. (2010). Low Risk of Colorectal Cancer and Advanced Adenomas More Than 10 Years After Negative Colonoscopy. Gastroenterology.

[11] R Core Team (2019). R: A Language and Environment for Statistical Computing.

[12] Hyun J.H., Kim S.J., Park J.H., Wie G.A., Kim J.-S., Han K.S., Kim B.C., Hong C.W., Sohn D.K. (2018). Lifestyle Factors and Bowel Preparation for Screening Colonoscopy. Ann. Coloproctol..

[13] Hassan C., Fuccio L., Bruno M., Pagano N., Spada C., Carrara S., Giordanino C., Rondonotti E., Curcio G., Dulbecco P. (2012). A Predictive Model Identifies Patients Most Likely to Have Inadequate Bowel Preparation for Colonoscopy. Clin. Gastroenterol. Hepatol..

[14] Lebwohl B., Wang T.C., Neugut A.I. (2010). Socioeconomic and Other Predictors of Colonoscopy Preparation Quality. Dig. Dis. Sci..

[15] Borg B.B., Gupta N., Zuckerman G.R., Banerjee B., Gyawali C.P. (2009). Impact of Obesity on Bowel Preparation for Colonoscopy. Clin. Gastroenterol. Hepatol..

[16] Schmilovitz-Weiss H., Weiss A., Boaz M., Levin I., Chervinski A., Shemesh E. (2007). Predictors of failed colonoscopy in nonagenarians: A single-center experience. J. Clin. Gastroenterol..

[17] Lee D.-W., Koo J.S., Kang S., Kim S.Y., Hyun J.J., Jung S.W., Yim H.J., Lee S.W. (2017). Association between bowel habits and quality of bowel preparation for colonoscopy. Medicine.

[18] Hendry P.O., Jenkins J.T., Diament R.H. (2007). The impact of poor bowel preparation on colonoscopy: A prospective single centre study of 10 571 colonoscopies. Color. Dis..

[19] Smith S., McGregor L., Raine R., Wardle J., Von Wagner C., Robb K. (2016). Inequalities in cancer screening participation: Examining differences in perceived benefits and barriers. Psycho Oncol..

