# Supplementary material for: Risk Factors of Inadequate Bowel Preparation for Screening Colonoscopy
Source: J Clin Med. 2021 Jun 21;10(12):2740. doi: 10.3390/jcm10122740 (PMC8233947; doi:10.3390/jcm10122740)
Supplement: Supplementary file 1 [file jcm-10-02740-s001.zip › jcm-1193357-supplementary.pdf]

Supplementary Table S1: **Previous studies assessing risk factors for inadequate bowel preparation.**

	First author/ Pub. Year/ Country	Recruitment years	Number of participants		Recruitment setting	Risk factors associated with poor bowel preparation (OR, 95%CI)
			Total	Inadequate bowel preparation		
**1**	**Hyun 2018, Korea** doi:10.3393/ac.2018.03.13 [11]	2012-2014	1079	422 (39.1%)	Screening	BMI > 25 (1.42, 1.11-1.85)
**2**	**Fayad 2013, USA** doi:10.1016/j.cgh.2013.05.037 [8]	2009	2163	956 (44%)	Screening (34.6%), surveillance (34.1%), diagnostic (31.3%)	BMI ≥30 (1.46, 1.21-1.75)<br>Use of tobacco (1.28, 1.07-1.54)<br>Narcotics (1.28, 1.04-1.57)<br>Hypertension (1.30, 1.07-1.57)<br>Diabetes (1.38, 1.12-1.69)<br>Dementia (3.02, 1.22-7.49)
**3**	**Hassan 2012, Italy** doi:10.1016/j.cgh.2011.12.037 [12]	2010	2811	925 (32.9%)	Screening (13.7%) and doctor referral	Liver cirrhosis (5.0, 2.6–10.4)<br>Parkinson disease (3.2, 1.2–9.3)<br>Diabetes (1.8 , 1.3–2.5)<br>Previous colorectal surgeries (1.6, 1.2–2.2)<br>Male sex (1.2, 1.02–1.5)<br>BMI *(1 point incr.)* (1.1, 1.03–1.1)<br>Age *(1 year incr.)* (1.01, 1.004–1.02)
**4**	**Lebwohl 2010, USA** doi:10.1007/s10620-009-1079-7 [13]	2006-2008 (endoscopy database query)	10,921*	2,389 (21.9%)	Screening (36%), diagnostic and surveillance	Age 70-79 (1.23, 1.07-1.42)<br>Male sex (1.44, 1.31-1.59)<br>Inpatient (1.51, 1.26-1.80)<br>After 11:00am (1.89, 1.71-2.09)
**5**	**Borg 2009, USA** doi:10.1016/j.cgh.2009.02.014. [14]	2007 (computerized record query)	1588	624 (39.3%)	Outpatient and inpatient. Screening and surveillance (58.4%)	BMI ≥25 (1.28, 1.01–1.61)<br>Male gender (1.36, 1.10–1.61)<br>Inpatient status (1.54, 1.11–2.13)<br>Smoking status (1.31, 1.03–1.67)<br>Antidepressant use (1.67, 1.22–2.29)<br>Narcotic use (2.06, 1.30–3.25)<br>Diabetes mellitus (1.37, 1.05–1.78)<br>Decreased mental capacity (2.17, 1.06–4.45)

*Lebwohl at al – number of participants with recorded quality of bowel preparation.
